# Improving Veteran Engagement with Virtual Care Technologies: a Veterans Health Administration State of the Art Conference Research Agenda

**DOI:** 10.1007/s11606-023-08488-7

**Published:** 2024-01-22

**Authors:** Taona P. Haderlein, Jenice Guzman-Clark, Navid S. Dardashti, Nicholas McMahon, Elizabeth L. Duran, Jolie N. Haun, Stephanie A. Robinson, Amanda C. Blok, Sarah L. Cutrona, Jan A. Lindsay, Christina M. Armstrong, Kim M. Nazi, Stephanie L. Shimada, Nancy R. Wilck, Erin Reilly, Eric Kuhn, Timothy P. Hogan

**Affiliations:** 1 VHA HSR&D Center for the Study of Healthcare Innovation, Implementation, & Policy, Los Angeles, CA USA; 2 Department of Veterans Affairs, Veterans Emergency Management Evaluation Center, Sepulveda, CA USA; 3 Southern Arizona VA Health Care System, Tucson, AZ USA; 4 NYU Grossman School of Medicine, New York, NY USA; 5 VA New York Harbor Healthcare System, New York, NY USA; 6 Center for Healthcare Organization and Implementation Research, VA Bedford Healthcare System, Bedford, MA USA; 7 Research and Development Service, James A. Haley Veterans Hospital, Tampa, FL USA; 8 Division of Epidemiology, Department of Internal Medicine, University of Utah, Salt Lake City, UT USA; 9 The Pulmonary Center, Boston University School of Medicine, Boston, MA USA; 10 VA Center for Clinical Management Research, VA Ann Arbor Healthcare System, United States Department of Veterans Affairs, Ann Arbor, MI USA; 11 Department of Systems, Populations and Leadership, University of Michigan School of Nursing, Ann Arbor, MI USA; 12 Department of Population and Quantitative Health Sciences, University of Massachusetts Chan Medical School, Worcester, MA USA; 13 Houston VA HSR&D Center for Innovations in Quality, Effectiveness and Safety, Michael E. DeBakey VA Medical Center, Houston, TX USA; 14 South Central Mental Illness Research, Education and Clinical Center (A Virtual Center), Houston, TX USA; 15 Baylor College of Medicine, Houston, TX USA; 16 Rice University’s Baker Institute for Public Policy, Houston, TX USA; 17 Connected Health Implementation Strategies, Office of Connected Care, Veterans Health Administration, Washington, DC USA; 18 Trilogy Federal, LLC, Arlington, VA USA; 19 KMN Consulting Services, LTD, Coxsackie, NY USA; 20 Department of Health Law, Policy and Management, Boston University School of Public Health, Boston, MA USA; 21 Division of Health Informatics and Implementation Science, Department of Population and Quantitative Health Sciences, University of Massachusetts Chan Medical School, Worcester, MA USA; 22 VISN 1 Mental Illness Research, Education, and Clinical Center (MIRECC), VA Bedford Healthcare System, Bedford, MA USA; 23 University of Massachusetts Chan Medical School, Worcester, MA USA; 24 National Center for PTSD, Dissemination and Training Division, VA Palo Alto Healthcare System, Menlo Park, CA USA; 25 Department of Psychiatry and Behavioral Sciences, Stanford University School of Medicine, Stanford, CA USA; 26 Peter O’Donnell School of Public Health, UT Southwestern Medical Center, Dallas, TX USA

**Keywords:** Vveterans, virtual care, telemedicine, eHealth, patient engagement

## Abstract

Although the availability of virtual care technologies in the Veterans Health Administration (VHA) continues to expand, ensuring engagement with these technologies among Veterans remains a challenge. VHA Health Services Research & Development convened a Virtual Care State of The Art (SOTA) conference in May 2022 to create a research agenda for improving virtual care access, engagement, and outcomes. This article reports findings from the Virtual Care SOTA engagement workgroup, which comprised fourteen VHA subject matter experts representing VHA clinical care, research, administration, and operations. Workgroup members reviewed current evidence on factors and strategies that may affect Veteran engagement with virtual care technologies and generated key questions to address evidence gaps. The workgroup agreed that although extensive literature exists on factors that affect Veteran engagement, more work is needed to identify effective strategies to increase and sustain engagement. Workgroup members identified key priorities for research on Veteran engagement with virtual care technologies through a series of breakout discussion groups and ranking exercises. The top three priorities were to (1) understand the Veteran journey from active service to VHA enrollment and beyond, and when and how virtual care technologies can best be introduced along that journey to maximize engagement and promote seamless care; (2) utilize the meaningful relationships in a Veteran’s life, including family, friends, peers, and other informal or formal caregivers, to support Veteran adoption and sustained use of virtual care technologies; and (3) test promising strategies in meaningful combinations to promote Veteran adoption and/or sustained use of virtual care technologies. Research in these priority areas has the potential to help VHA refine strategies to improve virtual care user engagement, and by extension, outcomes.

**Supplementary Information:**

The online version contains supplementary material available at 10.1007/s11606-023-08488-7.

## INTRODUCTION

Although evidence supports the effectiveness of specific virtual care technologies in specific care contexts,^1^ often these technologies are used less than intended to realize a desired outcome, or their use wanes over time.^2, 3^ Variations in uptake and use may attenuate the potential benefits of virtual care technologies. In alignment with the Veterans Health Administration (VHA) Office of Connected Care, we use the term “virtual care” to refer to health technologies intended to enhance the accessibility, capacity, quality, and experience of health care for Veterans, their families, and their caregivers, wherever they are geographically located. Examples of virtual care include but are not limited to telehealth services (e.g., synchronous video visits, asynchronous image delivery, remote patient monitoring), mobile health applications (apps), automated text message platforms, patient health portals (e.g., My Health***e***Vet), and wearable devices (e.g., activity trackers).

Engagement with virtual care technologies includes all of a user’s involvement with a specific technology, from uptake to sustained interactions.^4–6^ Without adequate digital access, one cannot engage with a virtual care technology, and without engagement, one cannot realize desired outcomes from the technology. We therefore define engagement as the decision to adopt *and continue using* a specific virtual care technology over time. Furthermore, it is important to recognize that what constitutes engagement can vary across different virtual care technologies.^7^ For example, a self-help app may be intended for active use over a defined period. In contrast, apps such as CBT-I Coach, an adjunct app to cognitive behavioral therapy for insomnia, are designed for use in tandem with a particular treatment.^8^ Similarly, an automated text messaging protocol may deliver a mix of motivational messages requiring only passive reading and occasional responses to assessment questions, while a chronic disease remote patient monitoring program may require daily interactions such as answering questions and submitting symptoms and vital sign information. Thus, “engagement” is a dynamic term that is best understood in relation to specific technologies and healthcare use cases.

**Figure 1:**
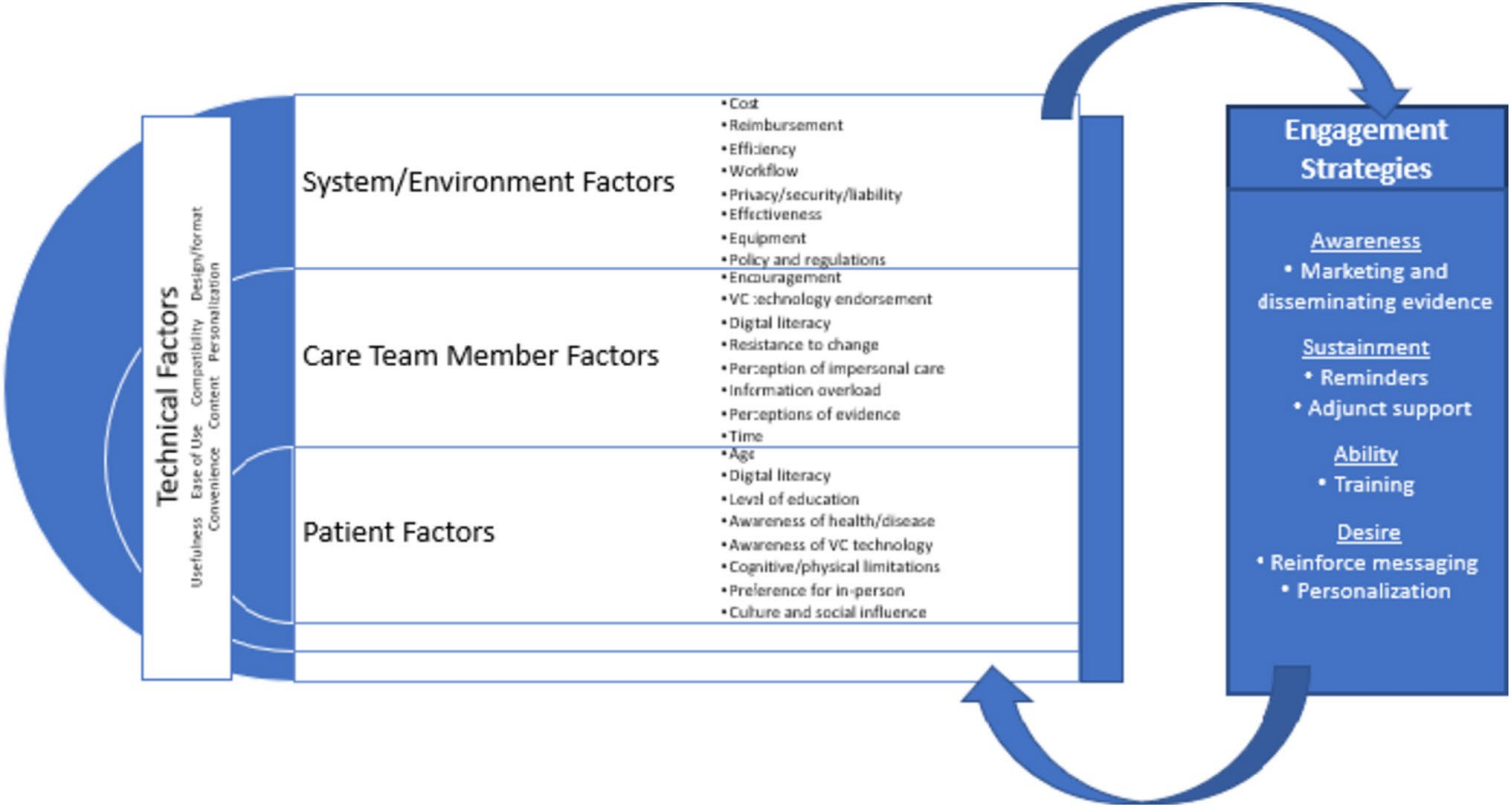
The virtual care engagement framework.

In Fig. 1, we introduce the Virtual Care Engagement Framework, which the present authors developed based on a review of the existing evidence. Factors at multiple levels can interact to influence Veteran virtual care engagement, including patient (e.g., age, health status/functioning, race, ethnicity, rurality),^9–13^ clinical team member (e.g., digital literacy, perceived burden, perceived value, proactive use of virtual care technologies),^14–17^ and system level factors (e.g., technology infrastructure, workflow, policy, and regulations).^2, 15, 18, 19^ Technical factors can vary by a technology or technology-assisted intervention itself, and may be cross cutting, requiring attention at different levels.^20^ Evaluating engagement is thus a complex endeavor. Strategies to increase virtual care engagement, such as adjunct support, training, and personalization, are designed to account for these factors. Conversely, some factors affect the use of these strategies depending on what is needed and who the strategy targets.

Healthcare organizations committed to the provision of high-quality virtual care, including VHA, have expressed the need for further investigation of these factors and of potential strategies that can be used to increase engagement. VHA’s Office of Connected Care (OCC) recognizes that the complex needs and risk factors of the Veteran population could impede Veteran engagement with virtual care technologies. In response, OCC designed and implemented various novel resources and innovative services across the VHA healthcare system to enhance engagement with virtual care technologies among different stakeholder groups. ^21^ These have included trainings, web-based video tutorials, help desk phone lines, toolkits, and Virtual Health Resource Centers within VHA facilities where Veterans and staff can receive training, hands-on support, and troubleshooting for use of different virtual care technologies.

Although increasing Veteran engagement with virtual care technologies is a VHA priority, research, clinical, and operations stakeholders lack consensus on a research agenda to support virtual care engagement and consequently, virtual care outcomes. Therefore, VHA Health Services Research & Development held a Virtual Care State of the Art (SOTA) Conference in May 2022 where separate workgroups convened to address research priorities for virtual care access, engagement, and outcomes. Here we report on findings from the engagement workgroup, including our workgroup processes, overarching workgroup discussion questions, key findings for each discussion question, and priorities for a research agenda on Veteran virtual care engagement in VHA.

## METHODS

### Participants

As stated above, the virtual care SOTA conference was organized into three workgroups, defined by focal areas specific to virtual care: (1) access, (2) engagement, and (3) outcomes. Details regarding the formation of workgroups and conference pre-work are available as part of this special journal issue. This article focuses on activities carried out by the engagement workgroup. The leadership committee of this workgroup (JG, TPH1, TPH2, ND, NM, ED) identified 14 experts through scholarly publications, virtual care research and clinical efforts in VHA, and related research funding. Because we aimed to develop a research agenda for improving Veteran virtual care engagement with VHA technologies, we sought VHA-specific expertise. We invited the experts we identified to serve as workgroup members. They represented a variety of career stages and organizational roles including researchers, clinical team members, administrators, and partners from VHA operational and clinical offices, including the Office of Connected Care and the National Center for PTSD. Representatives from these offices were invited because of their involvement in and/or commitment to the use of virtual care to meet their program objectives. The workgroup also included a Veteran representative who participated in defining the scope of the workgroup pre-conference and also attended the SOTA virtually.

### Pre-SOTA Activity: Identifying Key Questions

Leadership committee members developed a list of potential key questions to generate the research agenda. To ensure that workgroup activities yielded specific and actionable findings within the allotted time, the leadership committee limited the engagement workgroup’s focus to engagement with virtual care technologies among Veterans and their families and did include other VHA stakeholder groups (e.g., clinical team members). Through a consensus-building process, the committee ultimately identified three key questions for workgroup member consideration during the SOTA conference:

1. Based on the existing evidence about factors that influence engagement with virtual care technologies among Veterans, what additional research is needed to understand such factors?
2. Based on the existing evidence, what strategies at the Veteran, clinical team, and/or system levels show the most promise in supporting Veteran engagement with virtual care technologies?
3. What additional research beyond factors and strategies is needed to enhance Veteran engagement with virtual care technologies?

To ensure that workgroup members were similarly grounded in the existing evidence, including previous research both within and beyond VHA, as well as VHA initiatives to promote engagement with virtual care technologies, an overview and evidence brief was disseminated to the workgroup members prior to the conference (see Supplemental Appendix﻿). The brief featured a rapid review of published literature focused on factors that influence patient and Veteran engagement with virtual care technologies, relevant frameworks, strategies that could promote engagement, relevant VHA statistics, and information about related VHA programs.

### SOTA Conference Activities

The SOTA conference took place over the course of 2 days. Engagement workgroup members attended in person (*n*=12) or virtually (*n*=2). To support the deepest discussions possible, workgroup members were divided into three subgroups, with different subgroup member configurations to discuss each research question (3 subgroups for 3 questions, for a total of 9 configurations). On day 1, each subgroup participated in three sequential breakout sessions, one for each key question. The breakout sessions followed the same general procedure. The evidence brief was used to inform discussion and members were encouraged to identify research priorities for each key question. All discussions were audio-recorded and written materials produced during the sessions were collected and organized. Each breakout session was facilitated by a planning committee lead (TPH1, TPH2, JG) with an assigned notetaker. After eliciting feedback from other subgroup members to build consensus, the facilitator identified and synthesized the research priorities. The main priorities from each subgroup were then reported out to all engagement workgroup members. Next, each subgroup reconvened to review and revise the priorities. Finally, all engagement workgroup members reconvened, all identified research priorities were written on posterboard and hung on a wall, and each member independently assigned a ranking to each of the research priorities by placing a sticky note next to it with a number from one to five (the number one signifying the highest priority). To build consensus, the research priorities were then organized according to the rankings determined by the majority and based on the number of votes received and the frequency of no. 1 rankings. 

On day 2, leaders from the access, engagement, and outcomes workgroups presented their respective group’s findings to all SOTA Conference attendees. As a final activity, all SOTA attendees participated in a ranking exercise to identify the top research priorities across the access, engagement, and outcomes workgroups, as reported elsewhere in this special issue.

## Results

*Key Question 1: Based on the existing evidence about factors that influence engagement with virtual care technologies among Veterans, what additional research is needed to understand such factors?* Workgroup members discussed that a robust literature base exists on patient-level factors that influence engagement with specific virtual care technologies.^7, 9–11, 22–26^ However, less is known about the role of factors at clinical team, facility, or healthcare system levels.^12, 16, 17^ While some factors may be modifiable, the group noted that others may be less so. For example, age cannot be modified; however, strategies can be developed to address age-related changes that could affect engagement. It is also clear that some VHA clinical team members find it challenging to actively support Veteran engagement with virtual care technologies due to high workloads and limited time.^17^ Increased burden from the use of virtual care, whether actual or perceived, may negatively impact clinical team members’ willingness to use the technologies, reducing Veteran engagement with virtual care. ^27^

*Key Question 2: Based on the existing evidence, what strategies at the Veteran, clinical team, and/or system levels show the most promise in supporting Veteran engagement with virtual care technologies?* Workgroup members noted that although there is considerable existing research on factors affecting patient engagement with virtual care technologies,^7^ few studies have identified specific, successful strategies to improve it. Some studies suggest that at the individual patient level, provider endorsement and encouragement to use virtual care technologies, promoting awareness of the virtual care technologies available, and providing consistent technical assistance can improve patient virtual care engagement.^7, 22, 25, 26, 28–31^ At the broader clinic and/or system levels, local champions, internal facilitators, and leadership support for the role of virtual care technologies in patient care can facilitate patient virtual care engagement.^3, 32^ Additional research is needed to identify mechanisms of effective engagement strategies, and to develop and test novel interventions to improve engagement.

*Key Question 3: What additional research beyond factors and strategies is needed to enhance Veteran engagement with virtual care technologies?* Workgroup members described additional research needed in the following areas based on the discussions and ranking exercise:

1. The Veteran journey, including Veteran healthcare use and data across systems of care including but not limited to the Department of Defense (DoD) and VHA, how Veterans are introduced to virtual care technologies, and how easy it is for them to register, access, and use them.
2. Veterans’ healthcare priorities, goals, and desire for engagement with virtual care technologies during and after the COVID-19 pandemic.
3. The information and technology environments of Veterans more broadly, including their use of non-VHA virtual care technologies, their personal health information management practices, and how these factors may influence their engagement with virtual care technologies.
4. Appropriate engagement measures across virtual care technologies and use cases that are agreed upon and can be used consistently by the VHA research community.
5. How to promote a culture of trust and perceived value in virtual care technologies to the Veteran population despite ongoing change across the VHA healthcare system (e.g., the electronic health record modernization with Oracle Cerner®), the broader public health infrastructure, and the US healthcare system (e.g., the COVID-19 pandemic).
6. The role of informal caregivers (family, friends, peers) in promoting adoption and sustained use over time of virtual care technologies among Veterans.

### Priorities for Future Research

Discussion of the three key questions and attendees’ participation in a ranking exercise yielded the following list of top research priorities related to Veteran engagement with virtual care technologies. Table 1 lists examples of potential research questions to address each of these priorities:

1. *Understand the Veteran journey from active service to VHA enrollment and beyond, and when and how virtual care technologies can best be introduced along that journey to maximize engagement and promote seamless care*. Workgroup members noted that VHA has a unique opportunity to leverage Veterans’ institutional history with DoD to introduce VHA virtual care technologies during the transition between active service, discharge, and VHA enrollment. Although approved VHA clinical, operations, and research personnel can access both DOD and VHA data for some veterans, there is no shared virtual care platform that military personnel can access to manage their healthcare during both the active duty and post-active duty phases. During the SOTA, the workgroup members noted that shared patient-facing virtual platforms could facilitate the transition from DoD to VHA Care. Research is needed to determine when (e.g., early, mid-, late, or post active duty), by whom (e.g., VHA clinicians, administrators, peers), and how to present, promote, and sustain VHA virtual care technology use over time. This could involve introducing Veterans to VHA virtual care technologies through DoD technology platforms that they are already using and providing coaching or technical assistance for newly separated Veterans.
2. *Utilize the meaningful relationships in a Veteran’s life, including family, friends, peers and other informal or formal caregivers, to support Veteran adoption and sustained use of virtual care technologies*. VHA has long recognized the important role that informal caregivers (e.g., friends, family members, and peers) play in a Veteran’s life and engagement in their health care. Research has demonstrated that such individuals can increase patient engagement with healthcare services across varied clinical contexts.^33–35^ However, less is known about the role that informal caregivers can play to encourage and support Veterans’ digital literacy, adoption, and sustained use of virtual care technologies.
3. *Test promising strategies in meaningful combinations to promote adoption and/or sustained use of virtual care technologies*. As noted above, although an evidence base exists on factors that affect engagement with virtual care technologies, less research has demonstrated successful strategies to increase engagement. In comparison to the use of singular strategies, applying strategies in combination may more effectively impact Veteran engagement. Testing combinations of strategies that could potentially address barriers at different levels of analysis holds promise, as does differentiating strategies to support initial adoption from those that support sustained use. Workgroup members strongly agreed that a deeper understanding of strategies to promote both initial Veteran adoption of virtual care technologies and sustained use over time will be critical as virtual care technologies continue to evolve.
4. *Test strategies that can divert virtual care technology engagement tasks away from clinical team members*. Previous research has shown that VHA clinical team members often express feeling burdened by the need to support Veteran use of virtual care technologies. ^15, 17, 32, 34^ These clinical team members are facing high workloads and high risk for workplace burnout. A critical question is how best to leverage other strategies, including the further involvement of non-clinical VHA staff members (e.g., medical support assistants, clerks, administrators, VHA volunteer service members, and other integral personnel) and new technological advances such as artificial intelligence in the work of promoting Veteran engagement with virtual care technologies and providing needed support. Such research could design and test approaches (e.g., Virtual Health Resource Centers mentioned above) to offload specific tasks from clinical team members by integrating new strategies within collaborative care models while promoting the seamless integration of virtual care technologies into existing care delivery workflows.^36^
5. *Develop and disseminate measures of engagement that are appropriate for different virtual care platforms and use cases*. Workgroup members expressed that VHA research, operational, and clinical stakeholders currently lack adequate measures of engagement for different technology platforms and use cases. The development of appropriate engagement measures and their consistent application across virtual care technologies and use cases is needed to monitor, assess, and ultimately understand patterns of engagement among Veterans. Moreover, the consistent reporting of such measures, a shortcoming which has been identified in the existing literature, is crucial for improving virtual care engagement.^37^
6. *Characterize and evaluate the impact of clinical team, facility, and system-level factors on Veteran engagement with virtual care technologies*. As noted above, prior research has focused on patient-level factors that affect engagement with virtual care technologies. Although some knowledge exists regarding factors at broader levels of analysis (see Key Question no. 2), workgroup members agreed that the research community, and clinical and operational stakeholders, would benefit from research that fully characterizes multi-level factors relevant to patient engagement with virtual care technologies.
7. *Translate established strategies applied in other non-technology contexts to the domain of virtual care technologies*. Workgroup members noted that VHA has a history of implementing large-scale initiatives to improve Veteran engagement in healthcare services. For example, the VHA Whole Health transformation initiated a shift from focusing on episodic, disease-centered care to engaging and empowering patients throughout their lives to take charge of their life and health, emphasizing well-being and self-care along with conventional care and complementary and integrated health therapies.^38^ These previous implementation efforts provide a rich knowledge base that can be adapted to inform novel strategies applicable to bolstering Veteran engagement with virtual care technologies.

**Table 1:** Virtual Care Engagement Research Priorities

VHA virtual care engagement research priorities	Potential research questions
1. Understand the Veteran journey, from active service to VHA enrollment and beyond, and when and how virtual care technologies can best be introduced along that journey to maximize engagement.	• How can the military onboarding and discharge processes, including existing Department of Defense technology platforms, be leveraged to facilitate engagement with VHA virtual care technologies during the transition from active to post-active duty? • When, how, and by whom should virtual care technologies be introduced to promote continued use over time?
2. Utilize the meaningful relationships in a Veteran’s life, including family, friends, peers, and other informal caregivers, to support Veteran adoption and sustained use of virtual care technologies.	• How do significant others impact Veteran engagement with virtual care technologies? • How can significant others be included as collaborators/active participants in efforts to increase Veteran engagement with virtual care technologies?
3. Test promising strategies in meaningful combinations to promote adoption and/or sustained use of virtual care technologies.	• Are there specific combinations of empirically-supported strategies that can be used to optimize Veteran engagement with virtual care technologies?
4. Test strategies that can divert virtual care technology engagement tasks away from clinical team members.	• How can VHA incorporate non-clinical VHA staff members into existing clinical workflows to support Veterans’ use of virtual care?
5. Develop and disseminate context-sensitive measures of engagement appropriate for different virtual care platforms and use cases.	• How can VHA meaningfully measure virtual care engagement and sustained use? • Are there measures that can be used to assess engagement across different virtual care technology platforms?
6. Characterize and evaluate the impact of clinical team, facility, and system-level factors on Veteran engagement with virtual care technologies.	• Are there specific factors that impact Veteran engagement with virtual care technologies at the clinical team member, facility, and/or system levels?
7. Translate established strategies applied in other non-technology contexts to the domain of virtual care technologies.	• How can the VHA leverage institutional knowledge gained from prior large-scale implementation efforts to facilitate Veteran engagement with virtual care technologies?

### Limitations

The consensus and recommendations from the VHA virtual care SOTA engagement workgroup reflect the perspectives of VHA stakeholders who participated in the conference and therefore may not readily translate to other healthcare systems and non-VHA patient populations. The workgroup lacked representation from DoD which could have changed the breadth and depth of discussions as well as research priorities identified. Relatedly, although the work group included a Veteran representative who participated in the planning phase and attended the SOTA, Veterans were less involved in selecting key questions and research priorities. Given that little research exists on effective strategies to support engagement with virtual care technologies, we did not focus on policy recommendations; however, we acknowledge that change in policy will be necessary to implement our presented findings. In addition, we realize that diverse racial, ethnic, and cultural groups may have differing barriers and facilitators to engagement with virtual care that were not brought up during the discussions. Further research is needed to elucidate virtual care engagement experiences of diverse populations.

## CONCLUSIONS

Based on the consensus of leading VHA experts, this paper articulates a set of research priorities for bolstering Veteran engagement with virtual care technologies. As prior research has focused on documenting the various factors that impact initial patient engagement with virtual care technologies, future work should focus on designing and testing strategies to enhance continued engagement. Leveraging the Veteran journey from active service to VHA healthcare system enrollment and beyond, involving informal caregivers, and incorporating virtual care technologies into existing clinical workflows with the help of non-clinical staff show promise for optimizing Veteran engagement with virtual care technologies and are potentially high-impact domains for future research.

### Supplementary Information

Below is the link to the electronic supplementary material.

Supplementary file1 (PDF 1.10 MB)
