# Supplementary material for: Improving Veteran Engagement with Virtual Care Technologies: a Veterans Health Administration State of the Art Conference Research Agenda
Source: J Gen Intern Med. 2024 Jan 22;39(Suppl 1):21–8. doi: 10.1007/s11606-023-08488-7 (PMC10937853; doi:10.1007/s11606-023-08488-7)
Supplement: Supplementary file 1 — Supplementary file1 (PDF 1.10 MB) [file 11606_2023_8488_MOESM1_ESM.pdf]

VA Virtual Care Consortium of Research (VC CORE) State of the Art (SOTA) Conference

Pre-Conference Readings: Engagement Workgroup

Table of Contents

Evidence and Findings Brief

**Overview**	3
**Key Questions and Frameworks**	4
**Background/Context**	5
**Section 1:** Utilization of VC Modalities in VA	6
**Section 2:** Factors that Influence VC Engagement in VA	8
**Section 3:** Examples of Operational Initiatives to Increase VC Adoption and Engagement in VA	11
**Section 4:** Examples of Implementation Strategies to Increase VC Adoption and Engagement in VA Research and QI Projects	12
**Section 5:** Evidence from Systematic Reviews on Engagement with VC Outside VA	14
**References**	21
**Article 1:** Adoption of Mobile Apps for Depression and Anxiety: Cross-Sectional Survey Study on Patient Interest and Barriers to Engagement	23
**Article 2:** Barriers and Facilitators to the Use of e-Health by Older Adults: A Scoping Review	35
**Article 3:** Implementation Strategies to Enhance the Implementation of eHealth Programs for Patients with Chronic Illnesses: Realist Systematic Review	47

Thank you for participating in the VA Virtual Care (VC) SOTA. Your pre-conference assignment is to review this evidence brief and assigned readings focused on engagement with VC technologies. As you read the brief, we ask that you record your thoughts on the Key Questions below to help facilitate and enrich our in-person discussions. We also encourage you to compose additional discussion questions for the SOTA Conference.

During the SOTA, your workgroup facilitator(s) will lead the group through discussions toward the goal of reaching consensus on what is known (current evidence base) and what needs to be known (knowledge gaps) in key domains, thus allowing us to make and prioritize recommendations for future research related to VC. Following our discussions, workgroup leads will create a PowerPoint summarizing the discussion, agreed upon priorities, and recommendations, which will be presented to all SOTA participants on day two of the conference.

Overview of VA VC SOTA

The VA VC SOTA will focus on three areas, *access*, *engagement*, and *outcomes.* Figure 1 presents a conceptual framework for the SOTA, and illustrates the relationship between these three areas.

The overarching goal of the SOTA is to inform policy and clinical operations and generate a research agenda focused on opportunities to:

1. Address VC access disparities
2. Enhance Veteran engagement with VC
3. Define and improve outcomes influenced by VC

Figure 1. Conceptual Framework for VA VC SOTA

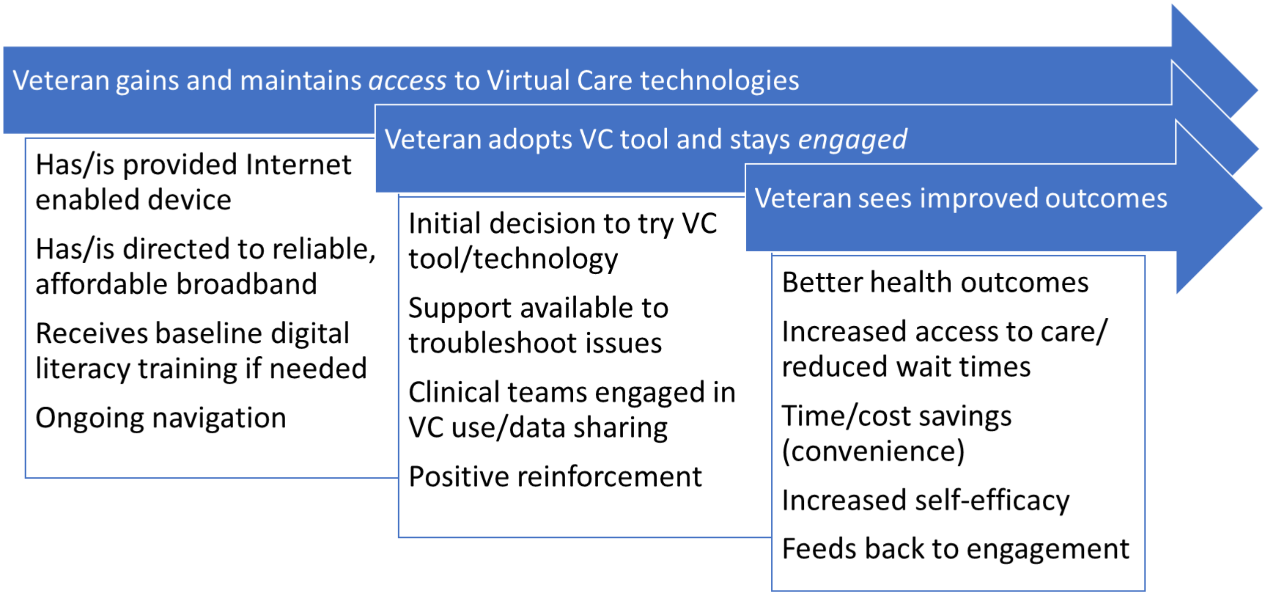

Key Questions and Frameworks for the Engagement Workgroup

The VC SOTA *Engagement* Workgroup will focus on the following key questions:

1. Based on the existing evidence about factors that influence engagement with VC among Veterans, what additional research is needed to understand such factors?
   a) **How should VA prioritize research related to these factors to maximize impact?**

2. Based on the existing evidence, what strategies at the Veteran, clinical team, and/or system-levels show the most promise in supporting Veteran engagement with VC?
   a) **How should VA prioritize research related to these strategies to maximize impact (e.g. research that will inform strategy design, testing, and policy)?**

3. What additional research beyond factors and strategies is needed to enhance Veteran engagement with VC?

As shown below, our SOTA engagement discussions will consider factors at the patient, care team, and broader system and environmental levels that can impact Veteran engagement with VC technologies, cross-cutting factors that are associated with VC technologies themselves, and examples of promising strategies that could support Veteran engagement with VC.

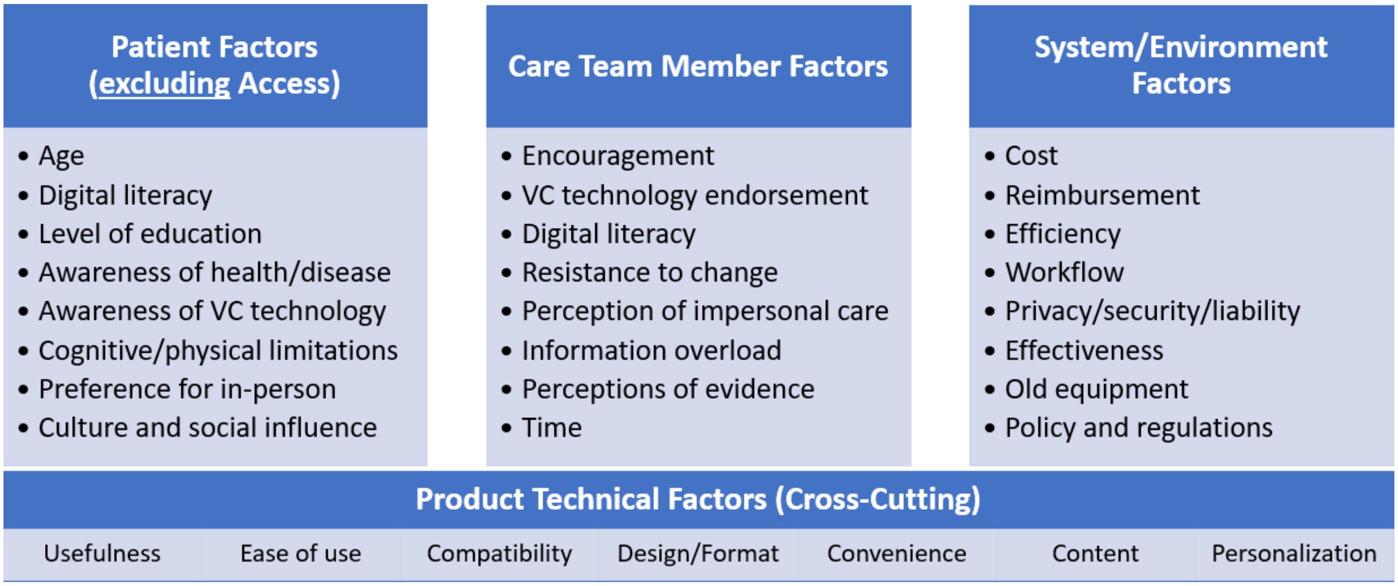

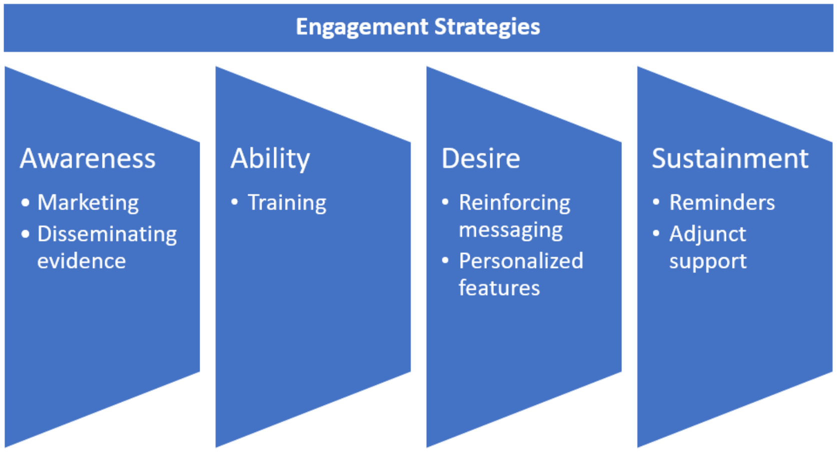

To inform our discussions, the Engagement Planning Workgroup conducted a rapid review of the published literature focused on factors that influence Veteran engagement with VC technologies, relevant frameworks, strategies that could promote engagement, and related interventions. Given its related focus, we also reviewed select literature from the field of implementation science regarding the adoption and use of VC technologies. We prioritized studies conducted within the VA health care system, but also examined evidence from outside VA that can inform the care of Veterans. The articles and ideas included in this document are not meant to be exhaustive, but rather, aim to provide a shared foundation for discussion. Below, we summarize some key themes and findings from this literature search.

Background/Context

What do we mean by “engagement” with VC technologies?

Although there is evidence for the effectiveness of using specific VC technologies in specific care contexts (e.g., using specific digital and mobile health (mHealth) apps for mental health conditions), it is often the case that individuals use these technologies less than intended, or have waning use over time. In implementation science terms, we might say that in such cases, the VC technology didn’t “stick” or that use was not sustained.

Variations in uptake and sustained use of VC technologies can have direct implications for realizing their intended benefits for the individual, their relationships with clinical team members, and the broader healthcare system. From such a perspective, “engagement” resides somewhere between digital access, our first SOTA working group, and outcomes, our third SOTA working group. Said differently, without digital access, one cannot engage with a VC technology; however, without adequate engagement, one cannot realize desired outcomes from the technology. As noted above, the charge to this group is to think about research related to user engagement with VC technologies.

To ensure we are starting from the same foundation, our group first needs a shared understanding of the term “engagement” for our work together. In the existing literature, user engagement with specific VC technologies has been described in different ways. For example:

- *User engagement refers to a user’s uptake and sustained interactions with a digital intervention, which includes interest in adopting an intervention…initial uptake …and continued use of an intervention*[1] (Borghouts et al, 2021)

- *The term ‘engagement’ refers to a user's involvement and interaction with a [digital] intervention* [2,3] (Arnold et al., 2021; Baltierra et al., 2016)

For our purposes in this workgroup, <u>we will characterize “user engagement” as a Veteran’s uptake and sustained used of specific VC technologies</u>. Because another SOTA workgroup is focusing on access, this workgroup will concentrate more on a Veteran’s decision to use and continue to use (rather than ability to use) VC technologies.

Further, it is also important that we recognize that what constitutes engagement varies across different VC technologies. While a self-help app may be intended for active use over a minimum six-month period, other apps intended to be used in conjunction with a clinical encounter may only be used in tandem with a treatment. An automated texting protocol may deliver a mix of motivational messages requiring only passive reading and occasional responses to assessment questions, while remote patient monitoring requires daily answers to questions and submission of daily vital signs for chronic disease monitoring. In these ways, engagement is a dynamic term and ties to specific technologies and use cases.

Section 1. Utilization of VC Modalities in VA

The purpose of this section is to broadly describe utilization of VA VC technologies across platforms. While these data show rapid growth in VC use for some technologies, engagement with VC still varies across technologies and subsets of the Veteran population.

1. **Shifts toward Synchronous Telehealth Use within the VA**
   In a national assessment of VC expansion in VA during the COVID-19 pandemic, Ferguson *et al.* showed large shifts in video-based, telephone, and in-person weekly encounters[4] (see Figure 1).

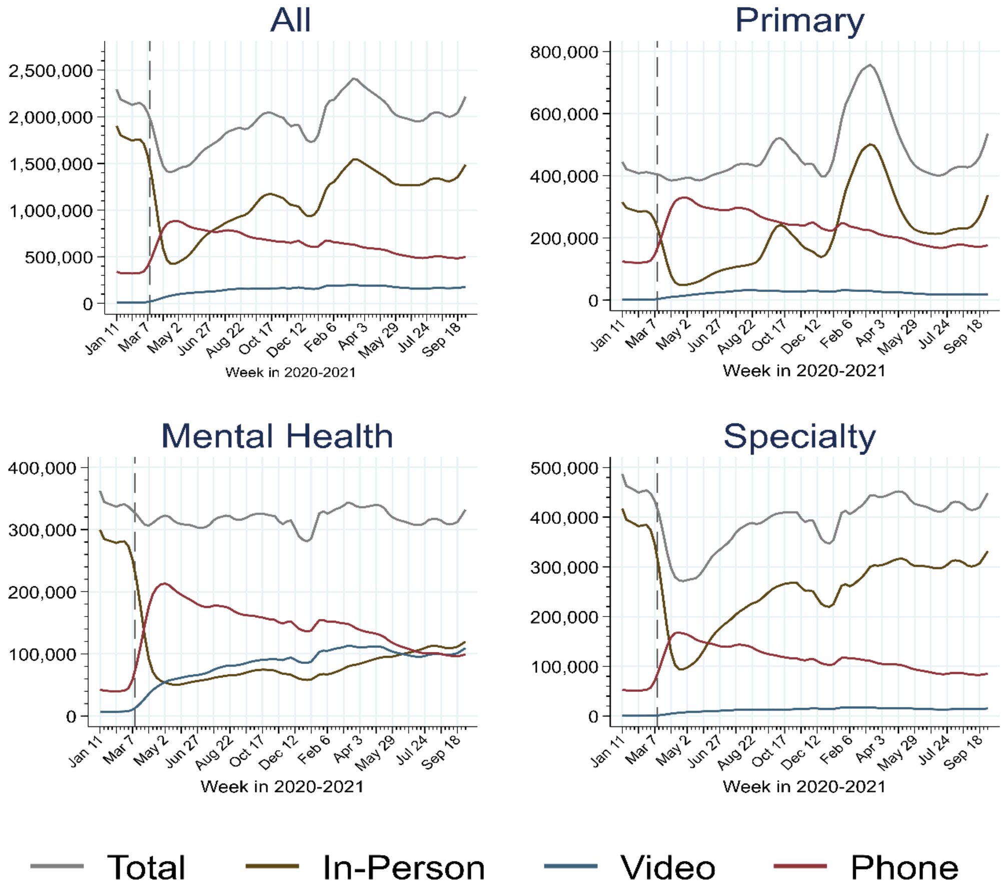

Figure 1: Encounters at the Veterans Health Administration (VA) between January 4, 2020 and October 2, 2021 by care delivery method and care type. The dashed line represents March 11, 2020: World Health Organization declares COVID-19 a pandemic. While all of primary care, mental health, and specialty care initially shifted toward telephone and video visits, mental health had the largest increase in video care and most sustained use of telephone and video.

   a. In FY21, approximately 1.9M unique Veterans completed over 9.5M VVC encounters. For comparison, VA has a population of approximately 6.5M patients.[5]
   b. In January 2022, nearly 474k patients completed a total of 829k VVC encounters.[5]
   c. Connolly *et al.,* 2021 found a stark difference in video visit use based on discipline, with psychologists having considerably higher video experience compared to psychiatrists, other medical providers (e.g., MDs and NPs), and other clinicians (e.g., social workers).[6]

2. **MyHealtheVet (MHV) Utilization Year over Year (YoY) and Cumulatively[7]**
   a. In January 2022, 1.51M unique MHV registrants (includes Veterans, healthcare teams, caregivers, and other beneficiaries) logged into MHV. Of these:
      - 671k Veterans and beneficiaries requested 2.71M prescription refills (+30% YoY and the most ever in one month).
      - 577k viewed appointments (+6% YoY) and received 3.5M email reminders.
      - 486k exchanged 2.8M secure messages with healthcare teams (+2% YoY).
      - 220k downloaded 600k files in Blue Button (the function of MHV that allows users to view and download information from their VA health record).

b. Cumulatively, MHV is the most used VC platform:
- 6.1M accounts created since November 2004; 3.6M authenticated Veterans
- 206M prescription refills requested since August 2005
- 150M secure messages initiated since June 2008

3. VA Web and Mobile App Utilization

a. VA Connected Apps (as of April 2022) – These apps connect to VA's network, so additional data security (e.g., secure login) is required to ensure protection meets federal standards. Data from connected apps can be seen by VA clinical team members. Utilization metrics for VA 'Connected' apps are also available on Power BI dashboards.

App (Web, iOS, & Android)	Cumulative Unique Users
VA Video Connect	2,271,589
Virtual Care Manager	139,379
Annie	46,417
VA Sync My Health Data	22,605
Mental Health Checkup	16,875
Patient Viewer	5,170
VA Pain Coach	3,333
Image Viewing Solution	2,493
My VA Images	1,467
VA Health Chat	838

b. VA Self-Contained Apps (more than 5 million total downloads) – These apps can be downloaded by anyone from Apple's App Store (iOS) or Google Play (Android), do not require any sign-in process and do not connect to the VA network. VA health care staff cannot see data in self-contained apps unless transmitted specifically by Veterans.

App (iOS & Android)	Downloads
Mindfulness Coach	1,001,087
CBT-i Coach	840,113
PTSD Coach	630,257
PFA Mobile	425,454
ACT Coach	302,936
PE Coach	288,115
AIMS	281,744
CPT Coach	267,401
COVID Coach	225,837
PTSD Family Coach	212,718
VetChange	189,990
Stay Quit Coach	189,614
STAIR Coach	175,409
Insomnia Coach	96,895
Couples Coach	70,503
Beyond MST	34,417

4. Home Telehealth/Remote Patient Monitoring (RPM)[5] – programs that apply care and case management principles to coordinate care using health informatics, disease management and technologies such as in-home and mobile monitoring, messaging and/or video technologies.

a. In FY21, approximately 150k Veterans used some form of RPM

<u>Section 2. Factors that Influence Implementation of and Engagement with VC in VA</u>

The purpose of this section is to summarize prominent factors impacting Veteran engagement with VC that have been documented in previous VA studies. Note that while the overall focus of the engagement workgroup is *Veteran* engagement with VC, we have also included some evidence regarding factors that influence VC use among VA clinical teams. Many VC modalities support some interaction between Veterans and their VA clinical teams, and clinician endorsement has been shown to be an influential factor in adoption of VC among Veterans and across VC platforms.[8-11] For these reasons, we anticipate that clinical team member engagement with VC will be a critical aspect of our group discussions. Of note, the engagement barriers listed in section 2 below report system-level barriers (IT, staffing) and provider-level barriers, and then focuses on more Veteran-level barriers.

1. **Research on Veteran and Provider Attitudes Toward the Use of Mobile Health Technology**
    a. In a study with interviews of 77 older Veterans, Gould and colleagues (2019)[12] found that:
        i. 65% of Veterans were found to prefer some form of technology (as opposed to printed materials), such as an app, website, or DVD, for self-management support
        ii. 29% indicated that an app was the first preference for delivery modality, and 35% preferred printed materials for self-management
    b. In a study of VA provider perceptions of mobile health, Miller et al (2019) found[13]:
        i. 91% of providers know how to find mobile apps and download them to a smartphone
        ii. 82% agreed “using apps helps to address my patients’ needs”
        iii. 81% “know other providers who have found value in using mobile apps”
        iv. 80% have “recommended using mobile apps to colleagues”
        v. 87% agreed “using apps makes it easier to provide educational materials”
    c. Customer feedback on VA ‘Connected’ apps are regularly pulled from many sources by VA’s Office of Connected Care (OCC), consolidated, then provided to the product teams for prioritization of updates
    d. Customer feedback for VA ‘self-contained’ apps are collected and consolidated by the team that develops those apps (mobilementalhealth@va.gov)

2. **Engagement Barriers at the System, Provider, and Patient Level**
    a. **IT Barriers**
        i. Usability/User interface issues
            1. Number of steps required for connection, authentication, and/or enrollment with a VC modality, including but not limited to virtual visits, automated texting, and the online patient portal[14,15,16]
            2. Inflexibility for using different types of VC modalities, including video-capable platforms (VVC)[14]
            3. Frequent logoffs, time required to log onto alternative systems (e.g., secure messaging through VA’s online portal)[17]
            4. Taken together, all of these points underscore that the usability of specific VC technologies can be a significant barrier
        ii. Functionality
            1. Inability to communicate with specific VA providers or care team members (as opposed to a Veteran’s larger care team)[14]
            2. Limited integration with VA’s EHR [17]
            3. Access for individuals other than the Veteran, including families and caregivers to messages[14]

    b. **Operational Barriers**
        i. Staff shortages and new workflows can result in VA clinical staff taking on multiple new roles which can be overwhelming[14]

ii. Limited clarity on staff roles and responsibilities can also present challenges in incorporating VC platforms into clinical processes[15]
iii. VA providers have cited structural and contextual barriers to mobile health adoption including not having time to discuss and assist with apps in care[13]
iv. VA providers have reported lack of resources for training Veterans and technical support for Veterans
v. Leadership support and clinic readiness can influence adoption and use over time[18]
vi. If there is not clarity and convergence, national policies related to VC can also be a barrier

c. **Digital Literacy and Training**
i. Clinical team members have described limited awareness of VC resources and not knowing how to integrate apps into clinical practice[13]
ii. There is a perceived lack of sufficient training among providers regarding VC modalities and needs for additional training[12,13]
iii. Limited awareness of VC resources among Veterans has also been noted in multiple studies[11,12]
iv. Lower computer proficiency among Veterans is associated with preferences for using different VC modalities (e.g., preferring DVDs over web and mobile apps)[12,19]
v. Insufficient knowledge of technical lexicon, menu symbols, limited comfort with technologies, and frustration with ongoing maintenance have been documented challenges for Veterans[12]
vi. Veterans face difficulties with passwords and log-ins.[16]
vii. VA clinical team members may assess Veteran suitability for VC modalities based on the Veteran's technology experience rather than potential benefits[18]

d. **Older Age**
i. Older age is associated with lower VC use among both Veterans[4,20] and clinical team members[6]
ii. Veterans over the age of 45 may be less likely to use certain VC modalities (e.g., video)[6]
iii. Older Veterans have been shown to be at higher risk of drop-out from VC programs (e.g., home telehealth)[21]
iv. Technology adoption among older Veterans may also relate to expectations of in-person social contact, suggesting that VC may be more appealing as a supplement, not substitute, for in-person care.[22]
v. Studies suggest older Veterans would benefit from simplified application designs and digital literacy training to increase comfort, confidence, and willingness to use.[23]
vi. The experience of homelessness is associated with more rapid physiological aging, suggesting age-related barriers may be more pronounced in this population.[22]
vii. Similarly, VA providers over the age of 50 have been shown to be significantly less likely to use certain VC modalities (e.g., video)[6]

e. **Racial Disparities and Racism**
i. Studies have shown that Black Veterans are less likely to use select VC technologies (e.g., VA's My Health***e***Vet patient portal).[20,22]
ii. Implicit bias on the part of healthcare workers and structural racism in the healthcare system may contribute to observable disparities.[22]
iii. Cultural tailoring of recruitment materials and outreach approaches can generate more interest in VC among specific racial and/or ethnic groups[22,24]

f. **Substance Use**

  i. Substance Use Disorder (SUD) has been associated with reduced likelihood of use for some VC modalities (e.g., video visits)[22]
  ii. Although there is some evidence that Veterans with SUD may prefer VC options like video to in-person visits, studies also indicate that Veterans actively using substances can have difficulty keeping video appointments, concentrating during visits, and express lower interest in interacting with healthcare providers via video[22]

g. **Effects of Health and Functional Status**
  i. Veterans with more chronic conditions have been found to be more likely to receive VC during the ongoing COVID-19 pandemic[4]
  ii. Other analyses have shown that Veterans with higher Care Assessment Need (CAN) scores and more functional impairment may be at higher risk of drop-out from VC (e.g., home telehealth) [21]
  iii. Functional limitations such as impaired vision or hearing, or large fingers can act as barriers to engagement.[12]
  iv. Symptom severity may result in lower use of some VC modalities (e.g., specific VA apps like the Concussion Coach)[25]

3. Evidence for Specific Clinical Specialties

a. **Mental Health**
  i. Clinician concerns, logistical obstacles, and technology itself have been identified as part of systematic reviews as prominent factors influencing use of select VC modalities (e.g., video telehealth)[26]
  ii. Specific diagnoses and service utilization has been shown to predict odds of VC use (e.g., use of the patient portal; use of video or phone versus in-person visits)[27]
  iii. Studies have also shown that limited proof of efficacy (71.8%), concerns about data privacy (59.1%), and not knowing where to find relevant apps (51.0%) are frequent concerns among Veterans when it comes to using VC modalities (e.g., apps) for mental illness needs[8]

b. **Primary Care**
  i. During the rapid mobilization of virtual primary care services in response to COVID-19, key barriers included workforce training, Veteran education, and insufficient technology infrastructure[28]
  ii. Experiences during the COVID-19 pandemic have underscored the important role that established scheduling practices can play in the use of VC modalities (e.g., video)[14]

c. **Specialty Care**
  i. Reach of VC training was more limited in specialty care clinicians compared to Primary and Mental Health care[29] leading up to the COVID-19 Pandemic [29]
  ii. Existing clinical workflows, including those related to scheduling, can result in perceived barriers to VC adoption (e.g., scheduling clerks not closely integrated into cardiology clinics result in a sense among cardiologists that scheduling is a barrier to telehealth adoption)[14]
  iii. In a survey among VA providers, subspecialty providers reported greater inabilities to conduct a required physical exam or ability to assess physical health status than primary care providers[6]

Section 3. Examples of Operational Initiatives To Increase VC Adoption and Engagement in VA

The purpose of this section is to briefly summarize select operational efforts within VA intended to support the initial adoption and use of VC technologies. As noted in each of the numbered sections, these efforts have targeted different VA stakeholders.

1. **Office of Connected Care (OCC) Strategies to Facilitate VC Expansion During COVID-19**
   a. **Training and support for the VA workforce[29]**
      i. Tactical areas that were critical to the VA's success include:
         1. Frontline clinicians directed to complete trainings
         2. Policy restrictions relaxed
         3. National helpdesk staffing was increased
         4. Local sites that dedicated highest numbers of staff to VC expansion were the most successful at onboarding Veterans
      ii. Increased access to training and materials
         1. Launched Connected Care Academy, a 'one-stop shop' for all VC training and 24/7 access to materials for VA healthcare staff and academic and other federal organizations that we partner with
         2. Launched the Office of Connected Care's Promotional Toolkit Directory (internal sharepoint directory of promotional materials)
      iii. Raise VA healthcare staff digital literacy
         1. Developed an 8-part 'VA Virtual Care Best Practices' TMS series
         2. Created clinical support tools, including Office of Connected Care Outreach Toolkit, for both Veterans and VA staff, available to the public
      iv. Standardization of practice
         1. VA now has a nationwide directive to incorporate a test call standard operating procedure into VVC workflow to ensure veterans are prepared for their VVC visit

   b. **Expanding the technology infrastructure**
      i. VA Clinical Resource Hubs provide a network of solutions that combine in-person care and telehealth support to underserved VA medical facilities. This allows rural Veterans to get the care they need, when they need it, regardless of location.
      ii. Launched new VA Virtual Health Resource Centers (VHRC) modeled after the original at the St. Cloud, MN VA to support Veterans, healthcare staff, and family members/caregivers on the use of VC tools and programs.
         1. In FY21 delivered over 5,000 consultations/visits
         2. In FY21 delivered a total of 383 training and marketing events to a total of 17,567 attendees (Veterans and VA staff) to increase awareness, knowledge, and skills in VC use (VHRC Power BI Data Dashboard)
         3. The development of additional VHRCs at VA facilities in FY22 has been included as part of National Director Performance Goals.
      iii. Launched the VHRC Implementation Consult Service (ICS) providing facilities the implementation roadmap, materials, and guidance needed to launch a VHRC
         1. As of February 2022, a total of 20 facilities/programs had requested consultation regarding building a VHRC; 4 facilities/programs began the VHRC ICS cohort process.
            a. VHRC ICS Leaf Request site
            b. VHRC Implementation Consult Service Team: VHAVHRC@va.gov
         2. Results and lessons learned when implementing VHRCs[30]:
            a. VHRC staff knowledge and skills of VC increased
            b. Staff and facility readiness to adopt VC increased
            c. Use of implementation best practices increased

d. VC utilization increased among Veterans and clinical team members
e. The establishment of VHRCs may increase efficiencies in delivery of VC training and consultation to staff and Veterans, which may increase capacity and decrease barriers to adoption.

c. **Providing consistent, multi-modal messaging to diverse stakeholders**
   i. A multi-modal communications plan was deployed to describe VA's virtual tools
   ii. Detailed instructions about accessing live helpdesk support and other messages especially aimed at new VC users were disseminated

2. **National Center for PTSD's Tech into Care Initiative**
   a. The National Center for PTSD (NCPTSD) created *Tech into Care* as an initiative to help anyone learn how to integrate technology into care for Veterans. The Program is focused primarily on the suite of mobile mental health apps created by NCPTSD.
      i. The apps provide self-help, education and support for PTSD and related issues. Health care professionals can use treatment companion apps with Veterans to keep them engaged between sessions. Most are available for iOS and Android devices.
      ii. Resources developed to support engagement with these apps include ongoing lecture series, app demonstration videos for both patients and clinicians, Continuing Education courses, and downloadable PDF guides and roadmaps, as well as other materials addressing, for example, privacy concerns and clinical information.
      iii. For Veterans who prefer to use a computer or who do not have a mobile device, NCPTSD has a variety of online courses.
      iv. The Practice-Based Implementation (PBI) Network hosts monthly interactive Tech into Care Community of Practice calls open to any VA staff.

Section 4. Examples of Implementation Strategies to Increase VC Adoption and Engagement in VA Research and QI Projects

The purpose of this section is to briefly summarize a few strategies that have been developed and tested as part of VA research or evaluation projects to increase adoption and continued use of select VC technologies. As with the content of this entire brief, Section 4 is not comprehensive and many members of the VA research community are pursuing work related to strategy development and testing for different VA stakeholders and contexts of care. It will be extremely helpful to talk about this body of work together in our group.

1. **Supported Adoption Program** – to encourage secure messaging (SM) use[16]
   a. Goals of this project included to (1) conduct a multisite, randomized, encouragement design trial to test the effectiveness of a supported adoption program (SAP) designed to increase patient engagement with the secure messaging feature of VA's online patient portal; and (2) evaluate the impact of the SAP and Veteran-level SM adoption.
   b. The SAP was comprised of multiple components, including 2 snail mailings sent to Veterans, 2 secure messages sent to Veterans from their VA primary care team's secure messaging account, and 1 telephone-based motivational interview with Veterans. The SAP components were developed to address key constructs of behavioral and motivational theories and reflected input from VA My HealtheVet Coordinators who regularly work with Veterans to facilitate patient portal access and SM use
   c. Compared to those Veterans who did not receive the SAP, those who received the SAP had significantly higher rates of SM adoption and SM use persisted a year after the encouragement ended. The adoption rate among SAP recipients was 24% at 21 months; 10% above the control group (usual care). Most adopters (70.3%) sent their first message without a motivational interview.

d. Veterans who received the SAP also noted higher perceived provider autonomy support and less telephone use to communicate with their VA provider compared to Veterans who did not receive the SAP.
e. Most common reported Veteran barriers to SM adoption include self-efficacy (not comfortable using computer), no perceived need, and difficulties with portal password and log-in.

2. **Augmented Implementation Facilitation** – E.g. for automated Texting System (aTS)[18]
   a. This project included a hybrid type 2 effectiveness implementation trial comparing two approaches for supporting implementation of an automated texting protocol via VA's Annie system (aTS) to support Veterans with Hepatitis C medication management
      i. Usual Implementation Clinics received the start-up experience that VA designed for all new clinics instituting the aTS: a live virtual demonstration and access to a resource website that included promotional materials and training guides. Usual Implementation clinics could receive troubleshooting assistance from the external facilitators (EFs) by phone or email, but only if and when they reached out to them.
      ii. In addition to the start-up experience above, Augmented Implementation clinics received an implementation toolkit, support for local champion development, and proactive outreach by the primary EF. The toolkit contained sections on evidence of texting in health care, suggestions for gaining leadership and clinic support, use of champions, tips and tools on how to use the aTS, and promotional materials to encourage clinic and patient participation. Proactive outreach from the EF included one in-person visit early in implementation efforts and check-ins with clinic champions throughout implementation.
   b. aTS reach and use was modest with 197 Veterans approached, 71 (36%) enrolled, 50 (25%) authenticated, and 32 (16%) using the aTS
   c. Providers found aTS appropriate with high potential for scale-up, but reported difficulties w/ startup, Veteran selection and recruitment, and clinic workflow integration
   d. Veterans generally found the aTS easy to use and helpful, but low perceived need for self-management support contributed to high declination to use the protocol.
   e. In augmented implementation facilitation clinics, more Veterans actively used the aTS HCV protocol compared with Veterans at usual implementation clinics
   f. Veterans who texted reported lower distress about failing HCV treatment & better adherence to HCV medication; sustained virologic response did not differ by group
   g. Facilitated implementation increased aTS engagement, but no between-group difference for clinical effectiveness outcomes

3. **Personalized Implementation of Video Telehealth (PIVOT)[31]**
   a. PIVOT is an implementation strategy used to increase adoption of video telehealth to home (VTH) across a large, urban VHA medical center (Houston VAMC)
   b. In PIVOT, a group of external facilitators (EFs) travels to an implementation site and undertakes several steps:
      i. Meet with health-system leadership and key stakeholders (e.g., information technology, MH leadership, site telehealth lead) to discuss nationally established, system-wide implementation goals; present evidence for VTH; and consult about where to initiate implementation efforts.
      ii. Identify on-site Internal Facilitators (IFs), often community or specialty clinic supervisors, with knowledge of the local system, influence, and existing relationships with providers. IFs are trained in VTH delivery and empowered to become a local VTH expert with support from EFs.
      iii. Identify Clinical Champion providers with help from IFs across clinics and disciplines (e.g., psychiatry, psychology, social work, masters-level counselors) to maximize

uptake. Ideally, one Clinical Champion is identified in each satellite community or specialty MH clinic where VTH implementation will occur.

iv. Train Clinical Champions in VTH delivery, then mentor and empower them to provide support and guidance to ensure consistent, positive VTH messaging.

v. provide support, resources (i.e. note templates, emergency guidance), and troubleshooting to help IFs create and sustain a VTH program.

vi. Throughout the implementation process, EFs review and compile multilevel (federal, state, local, organizational) best practices, ethical guidelines, laws, and mandates concerning VTH delivery, technology, and compensation.

vii. In preparation for sustainability, IFs are encouraged to take an active role in expanding and sustaining the innovation, with guidance from EFs on how to access information directly and to communicate with site leadership about relevant changes.

c. During its implementation from FY13 to FY18, the increase in the number of patients receiving VTH and VTH visits was 6.3 and 6.5 times (respectively) greater for Houston relative to median national improvement.

d. PIVOT-R, an adaptation for rural sites has also proven successful relative to the national median. Growth in number of Veterans treated by telehealth from FY18 to FY19 was 2.85 times greater than the national annual average of growth at VHA facilities.[32]

Section 5: Evidence from Systematic Reviews on Engagement with VC Outside VA

1. **Engagement with e-health in Older Adults[33]:** Systematic reviews have explored the benefits of e-health for older adults, finding clinically significant improvements in health behaviors (increased physical activity and healthy eating) as well as psychological and health outcomes (memory and blood pressure). A scoping review of barriers and facilitators to e-health engagement in this population found the following most frequently cited:
   - **a. Barriers:** lack of self-efficacy, knowledge, support, functionality, and information provision about the benefits of e-health
   - **b. Facilitators:** active engagement of the target end users in the design and delivery of e-health programs, overcoming concerns about privacy, support for enhancing self-efficacy in the use of technology, and integration of e-health programs across health services to accommodate the multimorbidity that older adults typically face
   - **c. Gaps:** Research on constructs of habit, hedonic motivation, price value, and social influence, inclusion of older adults in design process
   - **d. Findings** from this review suggested that older adults are more likely to use e-health services that are cognizant of their physical and functional needs, provide appropriate education and training to engage with e-health, address previous negative experiences of, and misconceptions about, digital health technologies; and employ strategies to enhance the perceived trustworthiness and credibility of e-health.

Table: Overview of findings from Barriers and facilitators to the use of e-health by older adults

Factor	Category	Barrier [# of studies]	Facilitator (# of studies)
Individual	Intrinsic	Aging limitations: reduction of hearing, sight, memory, fine motor control [5].<br>Perceived self-efficacy [4].<br>Lacking confidence in e-health [1].<br>Fear and dislike of technology [1].<br>No interest in learning [2].	Desire to learn [5].<br>Motivation to make a lifestyle change [2].<br>Altruism: wanting to contribute to scientific progress [3].
	Extrinsic	Lack of experience/skills with e-health [2] or technology [3].<br>Lack of knowledge of e-health [2].	Belief that e-health services are of benefit [4].<br>Convenience of e-health [1].

		Previous negative experience [1].<br>Unmet expectations [1].<br>Lack of need to change [4].<br>Fear traditional services may perish [1].<br>Disbelief in efficacy of e-health [3]<br>Lack of external accountability [2].<br>Inability to incorporate into routine [1].<br>Required effort [2].<br>Cultural limitations: language barriers and e-health detracting from time with family [1].	Ability to incorporate into current routine [3].<br>Previous experience and skills [4].<br>Previous experience with e-health and required skills [4].<br>Positive experience with technology generally [1].<br>Opportunity to learn new information [1].
Tech	Functional	Small screen and text [1].<br>Small icons, lack of color contrast [1].<br>Complex functionality [1].<br>Poor functionality [3].	Ease of use such as audio feedback, and large and clear visual display [4].
	Content	Lack of alerts [1].<br>Alert fatigue: reminders/emails/texts [1].<br>Condescending/impersonal communication [1].<br>Overwhelming and difficult to understand content [2].<br>Too much content on one page [1]	Personalized content [4].<br>Use of reminders/alerts [3].<br>Use of images [1].
Relational	Tech Support	No training/support to learn [2].<br>No one to help troubleshoot issues [1].<br>Reliance on family for guidance, and lack of family's patience and understanding while learning [1].	Training/support to learn [5].<br>Dedicated coach for training and continued support [1].<br>Peer-to-peer platform to share experiences [1].<br>Option for family/carer to support [1].
	Social Support	Lack of social interaction [2].<br>Absence of interpersonal communication [1].<br>Communication through technology considered an 'inauthentic experience' [1].	Socially inclusive and community-based information [1].
Organizational	Privacy	Health information concerns [3].	
	Trust	Unknown accuracy of information [3].<br>Knowing who are communicating with [1].<br>Concern in management of emergencies [1].<br>Concern over Western Medicine's prioritization of medication [1].	Recommendation from physician [2].<br>Content designed by experts in field [1].<br>Access to specialists in platform [1].<br>Authenticity: platform with clear credentials [1].
	Data sharing	Lack of communication between health platforms [1].	Sharing of health information between health care providers [3].

Table 1: Individual = persons' individual attributes including physicality, cognition, experience, skills, and knowledge; Technological = the use of the technology, including device functionality, content, and availability; relational = person-to-person engagement and support; Environmental = location context and characteristics; Organizational = structure, capabilities, and development of the service

2. **Patient Engagement with Digital Mental Health Interventions (DMHIs)** [1]– Defined as using digital formats such as smartphone apps, internet websites, wearable devices, virtual reality, or video games aimed primarily at a mental health target such as psychological well-being, anxiety, depression, stress, or mood. A systematic review classified barriers and facilitators of engagement with DMHIs into user, program, and technology/environment related constructs. The table below summarizes the findings of this review.

Category	Construct	Summary of findings
**User Related**	Demographic variables (sociodemographic factors, such as age, gender, and education)	Overall, women were more likely to engage with DMHIs than men
	Personal traits (factors related to personality traits, such as neuroticism and extraversion)	Neuroticism, agreeableness, openness, and resistance to change were associated with higher engagement, whereas extraversion was associated with lower engagement
	Mental health status (factors related to the current mental health status of the user, such as the type and severity of symptoms)	Severity of mental health symptoms increased the interest in DMHIs, but symptoms related to depression, mood, and fatigue were a barrier to actual engagement
	Beliefs (with regard to technology, mental health, and mental health services)	Positive beliefs about mental health help-seeking and technology-facilitated engagement
	Mental health and technology experience and skills (previous experience with technology, mental health technology and services, and skills related to digital, mental health, or digital health literacy)	Digital health literacy and positive experiences with mental health services and technology were facilitators to engagement
	Integration into life (the extent to which the user is able to find time and space to use the intervention and make it part of their routine)	Engagement was facilitated if people were able to integrate DMHI use into their daily lives
**Program Related**	Type of content (content and features offered by the intervention)	Engagement was facilitated if content was credible and if activities offered by the DMHI were of an appropriate length
	Perceived fit (factors related to how appropriate to the user's culture and values, and adaptable to the user's needs an intervention is; vs a one-size-fits-all solution)	Engagement was facilitated if information offered by a DMHI was customizable and relevant to the user
	Level of guidance (on how, when, how often to use the intervention, for example, through notifications or a coach)	Guided interventions, either through a human therapist or automated reminders to use a DMHI, had higher engagement than unguided interventions
	Social connectedness (the extent to which the intervention connects or isolates the user with or from others)	Being able to connect with other people through a DMHI facilitated engagement
	Impact of intervention on the user (such as an improvement or exacerbation of mental health symptoms [as measured by a validated survey scale])	DMHI engagement was facilitated if participants experienced a positive impact as a result of using a DMHI, such as the improvement of symptoms
**Technology and Environment related**	Technology-related factors such as the resources and costs required to use the intervention, usability, and technical issues experienced by the user)	Technical issues were a common barrier to engagement
	Privacy and confidentiality (factors related to data security, storage, confidentiality, and privacy of the digital intervention)	Engagement was facilitated if participants had a sense that the digital platform was private and anonymous, and they could safely disclose information
	Social influence (factors from the users' social environment, such as perceptions held by their peers, family, and health care provider, that influence their intention to use an intervention)	Participants were more likely to use DMHIs if people close to them, such as family and friends, thought they should use DMHIs

	Implementation (such as the availability of user training, the phase of the user's mental health care–seeking process during which the intervention is introduced or accessed, and characteristics of the health care organization supporting the DMHI)	DMHI engagement was facilitated if people were trained on how to use it

3. **Patient-Generated Health Data/Remote Measurement Technology[34] –** Defined as any mobile technology that enables monitoring of a person's health status through a remote interface, with the data then either transmitted to a health care provider for review or to be used as a means of education for the user themselves. A systematic review organized the barriers and facilitators most frequently noted as shown below.

   a. **Barriers:** Technical malfunctions were most widely reported (11 studies), including:
      i. Not receiving notifications or receiving them at the wrong time
      ii. Disappearance or freezing of the app, losing power or restarting without warning
      iii. Difficulties connecting remote (wearable and/or smart technology) devices with apps
      iv. Studies reported that this led to participant withdrawal, data loss, or significantly fewer data entries (e.g. by 35%)
   b. **Facilitators:** 4 studies demonstrated a positive and motivating effect of feedback.
      i. Buchem et al reported that 50% of participants felt motivated by virtual rewards such as badges (i.e. an indicator of accomplishment OR skill that can be earned)
      ii. Dale et al reported that 67% liked receiving motivational texts from the RMT system
      iii. Some participants reported a benefit associated with learning about their real-time activity [32] and talking about app data with a study coordinator
      iv. Further incentives that were suggested to increase motivation to engage included social sharing and comparison, or gaming features, including monetary rewards
      v. Another aspect reported to be "enjoyable" in 1 study was receiving the training instructions, which was seen to be an important contributor to increased engagement

Gaps: future research should focus on the entire engagement process and quantify the impact of specific variables on engagement in terms of observable changes in usage statistics in *rigorous experimental design*.

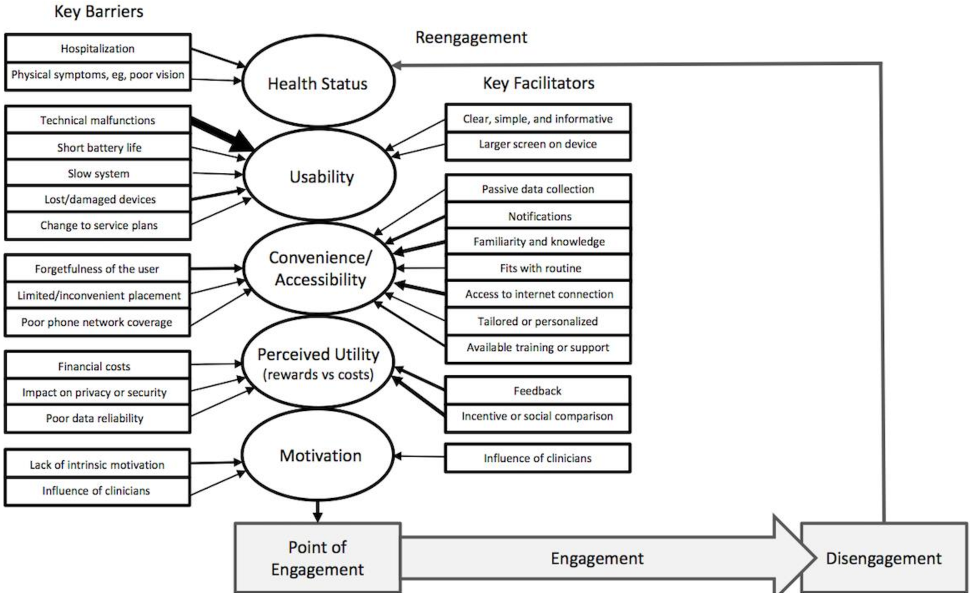

4. **Clinician Engagement with Mobile Health (mHealth) [35]:** Defined by WHO as "medical and public health practice supported by mobile devices, such as mobile phones, patient monitoring devices, Personal Digital Assistants (PDAs), and other wireless devices."

a. A systematic review identified various technical factors impacting clinicians' adoption of mHealth, summarized with their respective frequencies as shown below.

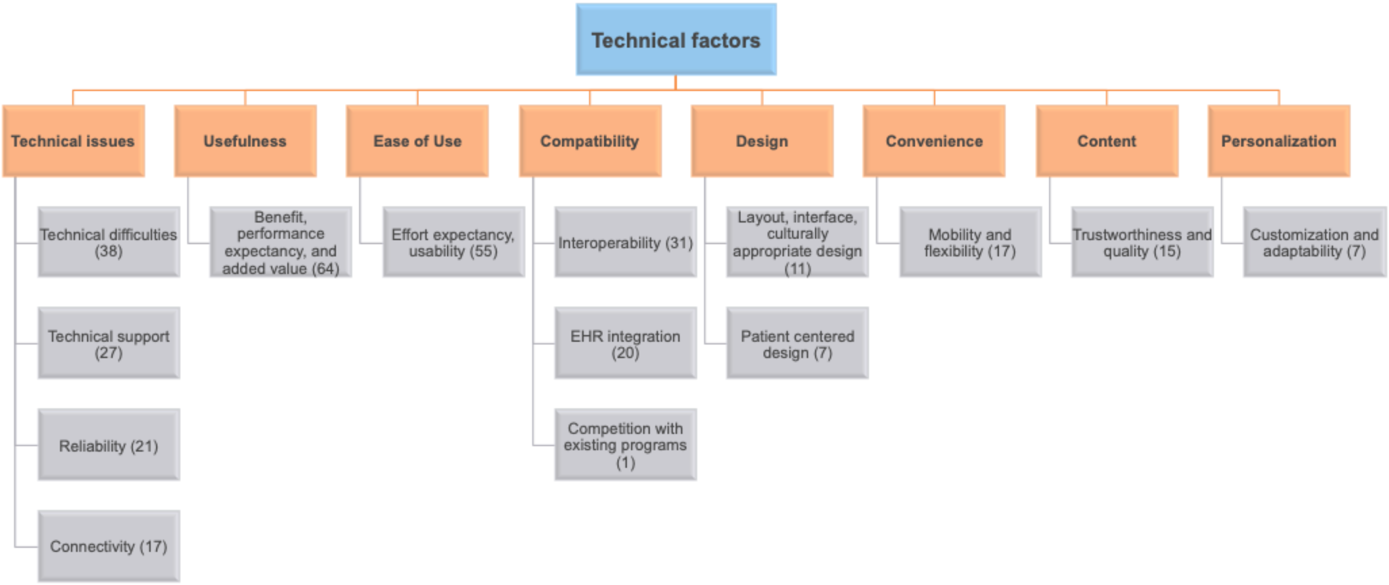

b. Social and organizational factors impacting clinicians' adoption of mHealth were manifestly more numerous than technical factors. These factors are summarized with their respective frequencies below.

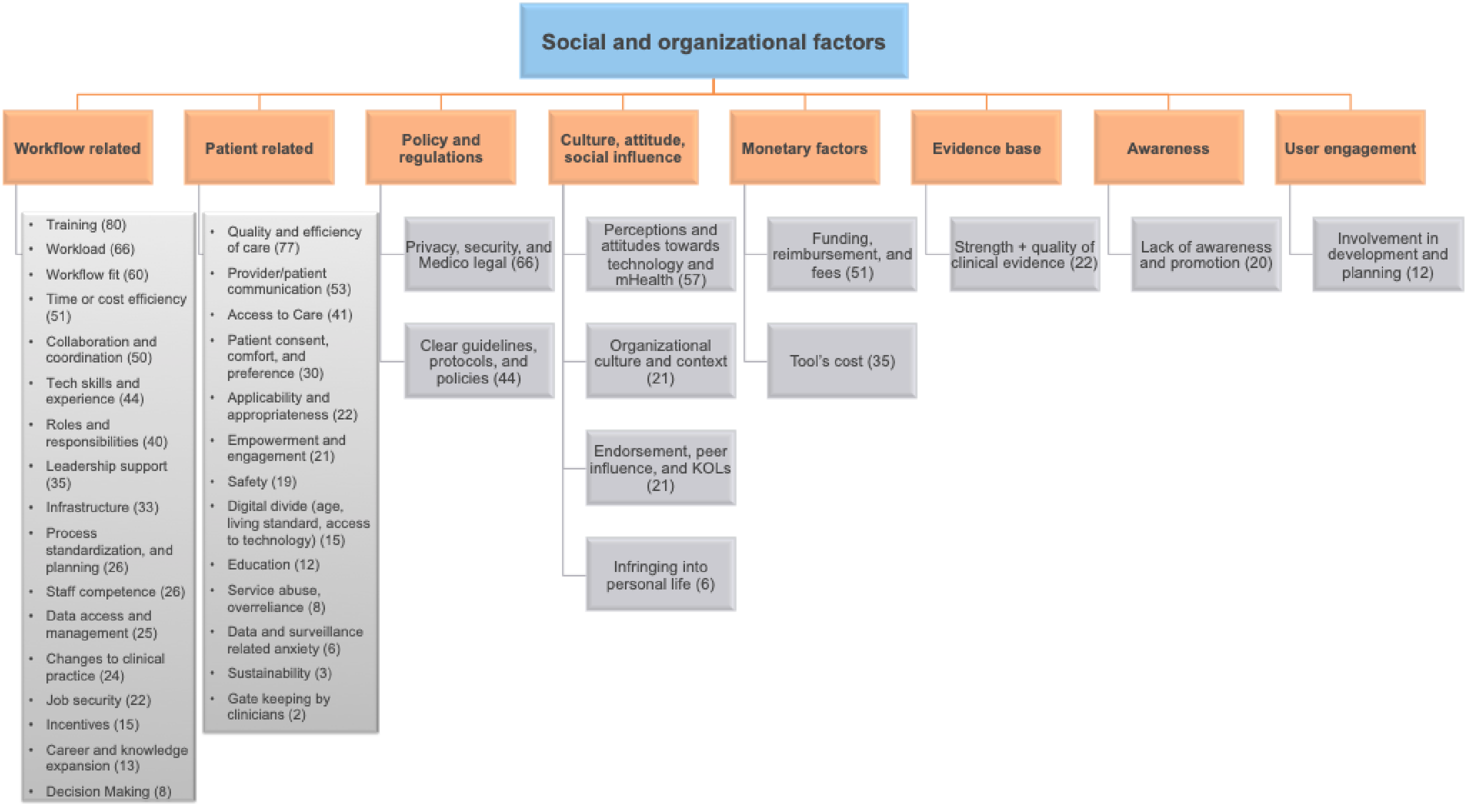

c. The review findings indicate important areas that must be targeted in future work to promote and foster clinicians’ successful adoption of mHealth tools.

POLICY

For **policy** makers

- Legislate regulations that simplify and facilitate **reimbursement**, and **data privacy and management**
- Incorporate mHealth in **health insurance** schemes
- Develop new **remote care protocols**
- Support the inclusion of mHealth-related skills in **medical education**
- Fund more research that contributes to a solid **evidence base**
- Harmonize to reach **interoperability**

DEVELOPMENT

For **mHealth** providers

- **Involve clinicians** in the design, planning, and implementation
- Facilitate **user feedback**
- Provide reliable **training material** about the tools’ features, benefits, and workflow integration scenarios
- Ensure continuous **technical support**
- Deliver **usefulness** and **ease of use**
- Work on solving **interoperability** and **EMR integration** issues
- Engage with **reliable clinical associations and opinion leaders** to endorse the tool and create trust

IMPLEMENTATION

For **clinical** decision makers

- Support a **cultural shift** that promotes the benefits of technology and innovation
- Facilitate mHealth **training programs**
- Integrate mHealth in **the workflow**
- Create **new roles** that support the implementation
- Encourage the creation of **multidisciplinary teams** combining digital and clinical expertise
- **Redefine the current roles** to reflect the new skills needed for a successful implementation

5. Implementation Strategies used in eHealth [36]

a. The goal of this systematic review was to summarize evidence from existing studies regarding implementation strategies used when implementing eHealth interventions for patients with chronic illnesses living at home, implementation outcomes, and the relationship between implementation strategies, implementation outcomes and degree of implementation success

b. A variety of electronic databases were searched and studies were included that described implementation strategies used to support the integration of eHealth interventions, and those strategies were categorized according to the Expert Recommendations for Implementing Change (ERIC) compilation of implementation strategies.

c. Implementation success was determined according to author reporting of implementation in the studies themselves. The review found that the following implementation strategies were directly related to implementation success in several studies:
   - Management support and engagement, internal and external facilitation, training, audit and feedback

d. The authors identified no clear relationship between the number of implementation strategies used and implementation success across the studies

e. The tables below summarize the studies included in this review and the components of their respective implementation strategies.

Overview of included studies

First author	Patient groups	eHealth	Setting	Implementation framework	Implementation stage	Study design	Data collection
Bailey	COPD[a]	Clinical monitoring	Sheltered housing	N/A[b]	Middle[c]	Case study (QUAL[d])	Self-report assessment, observation, focus groups, interviews, workshops
Boonstra	Long-term illnesses	Video consultation	Homecare	Structurationism	Middle	Single case study (MIXED)	Interviews, workshops, written reports, policy plans, meeting minutes, observations, quantitative data on system use
Fortney	Depression	ICBT[e], *Beating the Blues*, video consultation	Primary care safety net clinics	RE-AIM[g]	Early[h]	Quality improvement methods (QUAL)	Qualitative needs assessments
Hadjistavropoulos	Anxiety, depression	ICBT, *Wellbeing Course*	Community mental health clinics	CFIR[i]	Late[j]	Process evaluation (QUANT[k])	Online survey
Hendy	Long-term illnesses	Clinical monitoring, *WSD*[l]	Primary care trusts	NPT[m]	Late	Comparative, longitudinal, qualitative, ethnographic case study (QUAL)	Interviews, meeting observations, document review
Hendy	Long-term illnesses	Clinical monitoring, *WSD*	Health and social care organizations	N/A	Late	Longitudinal, ethnographic case studies (QUAL)	Observations, document review, informal discussions, interviews
Horton	COPD	Clinical monitoring	Homecare	N/A	Middle	Case study (QUAL)	Focus groups, field notes, meeting minutes
Lindsay	PTSD[n], anxiety, depression, insomnia, chronic pain, SUD[o]	Video consultation, *Video to Home*	VA[p] Medical Center	PARIHS[q], RE-AIM	Late	Mixed-method program evaluation (MIXED)	Interviews, quantitative data on system use
Taylor	COPD, chronic HF[r]	Clinical monitoring	Community health care	PDSA[s]	Middle	Case studies and action research methodologies (QUAL)	Workshop observations, focus groups, document review, field notes
Terpstra	Chronic pain	ICBT, *Master Your Pain*	Mental health care institutions	TDF[t], TAM[u]	Early	Descriptive design (QUANT)	Evaluation questionnaire
Wells	Chronic illness	Online PHR[v]	Health delivery organizations	N/A	Late	Grounded theory inductive approach (MIXED)	Interviews, Web-based survey
Wilhelmsen	Depression	ICBT, *Mood-GYM*	General practice	NPT	Late	Qualitative study (QUAL)	Telephone interviews

[a]COPD: chronic obstructive pulmonary disease; [b]N/A: not applicable; [c]Middle: 4-12 months postimplementation startup; [d]QUAL: qualitative; [e]ICBT: internet-delivered cognitive behavioral therapy; [f]EBP: evidence-based practice; [g]RE-AIM: reach, effectiveness, adoption, implementation, maintenance framework; [h]Early: 0-3 months postimplementation startup; [i]CFIR: consolidated framework for implementation research; [j]Late: >12 months postimplementation startup; [k]QUANT: quantitative; [l]WSD: Whole Systems Demonstrator; [m]NPT: normalization process theory; [n]PTSD: posttraumatic stress disorder; [o]SUD: substance use disorder; [p]VA: Veterans Affairs; [q]PARIHS: promoting action on research implementation in health services; [r]HF: heart failure; [s]PDSA: plan, do, study, act; [t]TDF: theoretical domains framework; [u]TAM: technology acceptance model; [v]PHR: patient health record; [w]GP: general practitioner.

Categories of implementation strategies used in the included studies

Studies	Engage consumers	Use evaluative and iterative strategies	Change infrastructure	Adapt and tailor to the context	Develop stakeholder interrelationships	Use financial strategies	Support clinicians	Provide interactive assistance	Train and educate stakeholders	Total categories reported	Overarching implementation strategy (authors' description)
Bailey	x		x						x	3	Training
Boonstra	x	x	x	x	x	x	x			7	Not reported
Fortney	x	x	x	x	x			x	x	7	External facilitation/mixed
Hadjistavropoulos	x	x			x	x	x	x	x	7	External facilitation
Hendy		x	x		x		x		x	5	Not reported
Hendy			x		x	x		x		4	Managerial strategies
Horton			x						x	2	Not reported
Lindsay		x	x	x	x			x	x	6	External facilitation
Taylor	x	x	x	x	x	x	x		x	8	Action research
Terpstra									x	1	Training
Wells	x	x		x	x	x	x		x	7	Mixed
Wilhelmsen									x	1	Training
**Total**	**6**	**7**	**8**	**5**	**8**	**5**	**5**	**4**	**10**		

References

1. Borghouts J, Eikey E, Mark G, et al. Barriers to and Facilitators of User Engagement with Digital Mental Health Interventions: Systematic Review. *J Med Internet Res.* 2021;23(3):e24387.
2. Arnold C, Farhall J, Villagonzalo K-A, Sharma K, Thomas N. Engagement with Online Psychosocial Interventions for Psychosis: A Review and Synthesis of Relevant Factors. *Internet Interventions.* 2021/09/01/ 2021;25:100411.
3. Baltierra NB, Muessig KE, Pike EC, LeGrand S, Bull SS, Hightow-Weidman LB. More Than Just Tracking Time: Complex Measures of User Engagement with an Internet-Based Health Promotion Intervention. *Journal of Biomedical Informatics.* 2016/02/01/ 2016;59:299-307.
4. Ferguson JM, Jacobs J, Yefimova M, Greene L, Heyworth L, Zulman DM. Virtual Care Expansion in the Veterans Health Administration During the Covid-19 Pandemic: Clinical Services and Patient Characteristics Associated with Utilization. *Journal of the American Medical Informatics Association.* 2020;28(3):453-462.
5. VHA Support Services Center (VSSC). Cvt Sft Telehealth Workload Cube. In: VHA Support Services Center (VSSC), ed2022.
6. Connolly SL, Stolzmann KL, Heyworth L, et al. Patient and Provider Predictors of Telemental Health Use Prior to and During the Covid-19 Pandemic within the Department of Veterans Affairs. *American Psychologist.* 2021.
7. Affairs UDoV. My Healthevet Product: Statistics. 2022; https://vaww.va.gov/MYHEALTHEVET/statistics.asp. Accessed 03/09, 2022.
8. Lipschitz J, Miller CJ, Hogan TP, et al. Adoption of Mobile Apps for Depression and Anxiety: Cross-Sectional Survey Study on Patient Interest and Barriers to Engagement. *JMIR Ment Health.* Jan 25 2019;6(1):e11334.
9. Hogan TP, Etingen B, McMahon N, et al. Understanding Adoption and Preliminary Effectiveness of a Mobile App for Chronic Pain Management among Us Military Veterans: Pre-Post Mixed Methods Evaluation. *JMIR Form Res.* Jan 20 2022;6(1):e33716.
10. Saleem JJ, Read JM, Loehr BM, et al. Veterans' Response to an Automated Text Messaging Protocol During the Covid-19 Pandemic. *Journal of the American Medical Informatics Association : JAMIA.* 2020;27(8):1300-1305.
11. Haun JN, Lind JD, Shimada SL, et al. Evaluating User Experiences of the Secure Messaging Tool on the Veterans Affairs' Patient Portal System. *J Med Internet Res.* Mar 6 2014;16(3):e75.
12. Gould CE, Loup J, Kuhn E, et al. Technology Use and Preferences for Mental Health Self-Management Interventions among Older Veterans. *International Journal of Geriatric Psychiatry.* 2020;35(3):321-330.
13. Miller KE, Kuhn E, Yu J, et al. Use and Perceptions of Mobile Apps for Patients among Va Primary Care Mental and Behavioral Health Providers. *Professional Psychology: Research and Practice.* 2019;50(3):204.
14. Der-Martirosian C, Wyte-Lake T, Balut M, et al. Implementation of Telehealth Services at the Us Department of Veterans Affairs During the Covid-19 Pandemic: Mixed Methods Study. *JMIR Form Res.* Sep 23 2021;5(9):e29429.
15. Yakovchenko V, McInnes DK, Petrakis BA, et al. Implementing Automated Text Messaging for Patient Self-Management in the Veterans Health Administration: Qualitative Study Applying the Nonadoption, Abandonment, Scale-up, Spread, and Sustainability Framework. *JMIR Mhealth Uhealth.* Nov 15 2021;9(11):e31037.
16. Shimada SL, Zocchi MS, Hogan TP, et al. Impact of Patient-Clinical Team Secure Messaging on Communication Patterns and Patient Experience: Randomized Encouragement Design Trial. *J Med Internet Res.* Nov 18 2020;22(11):e22307.
17. Ozkaynak M, Johnson S, Shimada S, et al. Examining the Multi-Level Fit between Work and Technology in a Secure Messaging Implementation. *AMIA ... Annual Symposium proceedings. AMIA Symposium.* 2014;2014:954-962.

18. Yakovchenko V, Hogan TP, Houston TK, et al. Automated Text Messaging with Patients in Department of Veterans Affairs Specialty Clinics: Cluster Randomized Trial. *J Med Internet Res.* Aug 4 2019;21(8):e14750.
19. Turvey C, Klein D, Fix G, et al. Blue Button Use by Patients to Access and Share Health Record Information Using the Department of Veterans Affairs' Online Patient Portal. *Journal of the American Medical Informatics Association : JAMIA.* Jul-Aug 2014;21(4):657-663.
20. Abel EA, Shimada SL, Wang K, et al. Dual Use of a Patient Portal and Clinical Video Telehealth by Veterans with Mental Health Diagnoses: Retrospective, Cross-Sectional Analysis. *J Med Internet Res.* Nov 7 2018;20(11):e11350.
21. Guzman-Clark J, Farmer MM, Wakefield BJ, et al. Why Patients Stop Using Their Home Telehealth Technologies over Time: Predictors of Discontinuation in Veterans with Heart Failure. *Nurs Outlook.* Mar-Apr 2021;69(2):159-166.
22. Garvin LA, Hu J, Slightam C, McInnes DK, Zulman DM. Use of Video Telehealth Tablets to Increase Access for Veterans Experiencing Homelessness. *J Gen Intern Med.* Aug 2021;36(8):2274-2282.
23. Sedlander E, Barboza KC, Jensen A, et al. Veterans' Preferences for Remote Management of Chronic Conditions. *Telemedicine and e-Health.* 2018/03/01 2017;24(3):229-235.
24. Armstrong CM, Ortigo KM, Avery-Leaf SN, Hoyt TV. Cultural Considerations in Using Mobile Health in Clinical Care with Military and Veteran Populations. *Psychological Services.* 2019;16(2):276.
25. Bradley SE, Haun J, Powell-Cope G, Haire S, Belanger HG. Qualitative Assessment of the Use of a Smart Phone Application to Manage Post-Concussion Symptoms in Veterans with Traumatic Brain Injury. *Brain Inj.* Jul 2 2020;34(8):1031-1038.
26. Muir SD, de Boer K, Nedeljkovic M, Meyer D. Barriers and Facilitators of Videoconferencing Psychotherapy Implementation in Veteran Mental Health Care Environments: A Systematic Review. *BMC Health Serv Res.* Nov 1 2020;20(1):999.
27. Etingen B, Hogan TP, Martinez RN, et al. How Do Patients with Mental Health Diagnoses Use Online Patient Portals? An Observational Analysis from the Veterans Health Administration. *Adm Policy Ment Health.* Sep 2019;46(5):596-608.
28. Reddy A, Gunnink E, Deeds SA, et al. A Rapid Mobilization of 'Virtual' Primary Care Services in Response to Covid-19 at Veterans Health Administration. *Healthc (Amst).* Dec 2020;8(4):100464.
29. Heyworth L, Kirsh S, Zulman D, Ferguson JM, Kizer KW. Expanding Access through Virtual Care: The Va's Early Experience with Covid-19. *NEJM Catalyst Innovations in Care Delivery.* 2020;1(4).
30. Armstrong CM, Wilck NR, Murphy J, et al. Results and Lessons Learned When Implementing Virtual Health Resource Centers to Increase Virtual Care Adoption During the Covid-19 Pandemic. *Journal of Technology in Behavioral Science.* 2021:1-19.
31. Lindsay JA, Day SC, Amspoker AB, et al. Personalized Implementation of Video Telehealth. *Psychiatric Clinics.* 2019;42(4):563-574.
32. Day SC, Day G, Keller M, et al. Personalized Implementation of Video Telehealth for Rural Veterans (Pivot-R). *Mhealth.* 2021;7.
33. Wilson J, Heinsch M, Betts D, Booth D, Kay-Lambkin F. Barriers and Facilitators to the Use of E-Health by Older Adults: A Scoping Review. *BMC Public Health.* 2021/08/17 2021;21(1):1556.
34. Simblett S, Greer B, Matcham F, et al. Barriers to and Facilitators of Engagement with Remote Measurement Technology for Managing Health: Systematic Review and Content Analysis of Findings. *J Med Internet Res.* 2018/07/12 2018;20(7):e10480.
35. Jacob C, Sanchez-Vazquez A, Ivory C. Social, Organizational, and Technological Factors Impacting Clinicians' Adoption of Mobile Health Tools: Systematic Literature Review. *JMIR Mhealth Uhealth.* 2020/2/20 2020;8(2):e15935.
36. Varsi C, Solberg Nes L, Kristjansdottir OB, et al. Implementation Strategies to Enhance the Implementation of Ehealth Programs for Patients with Chronic Illnesses: Realist Systematic Review. *J Med Internet Res.* 2019;21(9):e14255.
